# Simultaneous Evaluation of Creep Deformation and Recovery of Bulk-Fill Dental Composites Immersed in Food-Simulating Liquids

**DOI:** 10.3390/ma11071180

**Published:** 2018-07-10

**Authors:** Ali Alrahlah, Rawaiz Khan, Khalid Alotaibi, Ziad Almutawa, H. Fouad, Mohamed Elsharawy, Nikolaos Silikas

**Affiliations:** 1 Restorative Dental Sciences Department, College of Dentistry, King Saud University, Riyadh 11545, Saudi Arabia; 2 Engineer Abdullah Bugshan research chair for Dental and Oral Rehabilitation, College of Dentistry, King Saud University, Riyadh 11545, Saudi Arabia; krawaiz@ksu.edu.sa; 3 Dental intern, College of Dentistry, King Saud University, Riyadh 11545, Saudi Arabia; khaledotaibi92@gmail.com (K.A.); ziyad1993@gmail.com (Z.A.); 4 Applied Medical Science Department, Community College, King Saud University, Riyadh 11545, Saudi Arabia; menhfef@ksu.edu.sa; 5 Department of Biomedical Engineering, Faculty of Engineering, Helwan University, Helwan 11792, Egypt; 6 Dental Biomaterials Research Chair, King Saud University, Riyadh 11433, Saudi Arabia; eng.melsha3rawy@yahoo.com; 7 Dentistry, School of Medical Sciences, University of Manchester, Manchester M13 9PL, UK; nick.silikas@manchester.ac.uk

**Keywords:** bulk-fill, viscoelastic properties, creep, resin-composite, food-simulating organic solvents, dynamic mechanical analyzer

## Abstract

The aim of this study is to compare the creep/recovery behavior of bulk-fill dental composites after storage in various food simulating organic solvents. For this purpose, five different resin-composites (four bulk-fills and one conventional) were used. A total of 20 rectangular specimens (14 mm × 3 mm × 0.7 mm) were prepared by filling the resin-composites in Teflon mold. All of the specimens for each material (*n* = 5) were divided into four groups namely dry (control), distilled water (DW), artificial saliva, and absolute ethanol. The specimens were subjected to three-point bending creep test during immersion directly. A constant load of 2 N was used for each specimen with loading and unloading time 2 h each. Results: SF2 and XF showed a lower creep strain % after immersion, ranging from 0.44 (dry) to 0.75 (saliva) and 0.43 (dry) to 0.80 (ethanol), respectively. TNC BF depicts the maximum creep strain % ranging from 1.24% (dry) to 2.87% (ethanol) followed by FBF ranging from 1.17 (dry) to 2.59 (ethanol). However, the conventional material (GR) showed lower creep strain after immersion ranging from 0.28 to 0.54. Moreover, SF2 resulted in the highest creep recovery in all of the composites groups, as well as conventional material. The other composite groups showed lower creep recovery as compared to the conventional material (GR). The creep strain % for all the bulk-fill composites materials were increased during immersion in the liquids. However, for the conventional material, the creep deformation is decreased after immersion. SF2 showed the highest percentage of creep recovery among the bulk-fill composites, followed by XF.

## 1. Introduction

Nowadays, resin-composite restorations are considered by many dentists as the first option in restoring carious lesion [1]. Nonetheless, they still have some limitations. The depth of cure is one of these limitations, which can affect the physical and biological properties of the restoration adversely. Therefore, it is recommended to use a layering technique by applying 2 mm thick oblique increment for each layer. In the last few years, bulk-fill resin-composites have been introduced, which can be applied in 4–5 mm layers, according to manufacturer instructions [2,3]. Two types of bulk-fill composites are available in the market: flowable bulk-fill composite that must be followed with a final layer of at least 1.5 mm to be filled with conventional composite; and, the regular bulk-fill composite that can be used to restore the whole cavity [4,5,6].

Clinically, resin composite restorations are exposed to temperature changes, chemical agents that are found in saliva, food, and beverages [7]. It has been reported previously [8,9] that chemical agents in food and beverages can reduce surface hardness of enamel, dentine, microfilled composite, resin modified glass ionomer, and also affect the viscoelastic properties. The reduction in surface hardness of microfilled composites may lead to some dimensional changes, including creep deformation. The measurement of viscoelastic properties can help to assess the propensity of the material to creep under load. Creep deformation and recovery are dependent on the material composition and storage media [10], and it will adversely affect the longevity of the restoration because the mechanical stress resistance will be affected [10,11].

Several studies showed that bulk-fill composites have an acceptable range of creep deformation and recovery when compared with conventional resin-composites [11,12,13,14]. As previously reported, flowable bulk-fill showed higher efficiency in curing depth when compared to regular bulk-fill but regular bulk-fill showed higher creep resistance than flowable bulk-fill [15]. It has been demonstrated that water and oral fluids significantly increased the creep deformation in resin-composites by detaching the filler from the matrix [14].

Chemicals in the oral cavity will cause surface the softening and roughening of restorations. Different food simulating organic solvents (FSOS) have different effects on resin-based restoration components. For example, the coupling agent can be disintegrated by oral fluids, the resin-matrix can be softened by organic acids, various food and liquids, and alcohol, while inorganic filler could be damaged by weak acids and water [16].

Three-point creep bending nano-indenter or uniaxial bulk compression devices measure creep deformation and recovery. The uniaxial compression consists of a rigid base of stainless steel, a cylindrical platform of stainless steel immersed in a water bath, a temperature controller, loading arm, and loading rod. It measures the creep of a specimen by applying a continuous load to it for a certain period of time, and measures recovery by unloading the specimen for the same period of time [10,14,17].

In this study, three-point creep bending test is evaluated by a dynamic mechanical analyzer (DMA) was used. It measures creep by holding the specimen from two ends and applying a controlled force on its middle for a certain period, and it measures the recovery by unloading the force over the same period. Apart from dry samples, all of the testing was completed while immersed in food simulating solvents in a special bath at 37 °C.

Previous studies reported that the viscoelastic behavior, especially creep of dental composites, has been influenced by many factors, such as filler composition, resin-matrix interaction, and degree of conversion [18,19,20]. However, interaction with food-simulated solvents can reduce the strength and modulus of the material, and thus may also affect creep [21]. Therefore, the aims of this study was to evaluate the viscoelastic stability of resin based dental composites by creep and recovery analysis, after immersing in food-simulating solvents. The null hypotheses were that the monomer type/resin matrix of resin-composite, type, and the amount of filler content have no effect on creep. Also, food-simulating solvents have no effect on creep of resin-composites.

## 2. Materials and Methods

Five commercial resin based composite materials, with varying matrix composition and filler loading, were used in order to study the effect of various FSOS on viscoelastic properties of bulk fill and conventional nano hybrid resin composites. The composition of bulk-fill materials, manufacturer increment thickness, and resin type for the materials that were used in this study are provided in Table 1. Four bulk-fill resin-composites, namely Tetric-N-Ceram, FiltekTM, SonicFill2TM, X-tra fil, and Grandio nano-hybrid resin-composite (control), were evaluated for creep deformation and recovery. Twenty rectangular specimens of 14 mm × 3 mm × 0.7 mm were prepared in a Teflon mold contained within a ring made of resin. The resin-composite was packed in the mold carefully, then a Mylar strip was positioned against the composite by a top plate. The mold was light cured, according to the manufacturer’s instructions. Specimens were divided into four groups (*n* = 5 each), as follows: Group 1, dry (control); Group 2, distilled water (DW); Group 3, (artificial saliva); and, Group 4, (absolute ethanol).

To prepare the artificial saliva, 100 mL of KH_2_PO_4_, Na_2_HPO_4_, HKCO_3_, NaCl, and MgCl_2_ + 6H_2_O were mixed, 8 mL of citric acid was added, followed by10 mL of CaCl_2_. The solution was diluted with distilled water to a final volume of 100 mL [22]. The pH range of the final solution was measured by pH meter and was recorded between 6.7 and 7.3. The ingredients and their concentration are given in Table 2.

**Figure 1:**
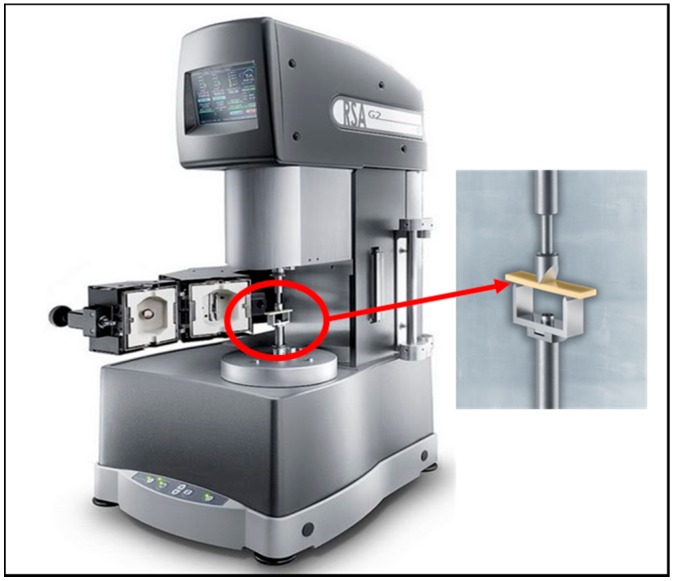
Dynamic mechanical Analyzer for three-point bending creep Analysis.

The creep test was conducted using a dynamic mechanical analyzer (TA instruments; New Castle, DE, USA), (RSA-G2 Solids Analyzer: Figure 1) under three-point bending mode. A Netzsch three-point bending sample holder with a span of 10 mm was employed. Each sample was prepared and immersed in its medium, and then immediately subjected to creep test. The test time set for deformation was 7200 s at a constant load of 2 N and 7200 s for recovery at a minimum load of 0.2 N (total time to complete test was 4 h, 2 h creep, and 2 h recovery). If the creep failure of specimens was performed in the examined time, the test finished immediately.

Statistical analysis was carried out using SPSS version 21.0 (SPSS Inc., Chicago, IL, USA). The maximum creep deformation and maximum recovery data were statistically analyzed using analysis of variance two-way ANOVA, one-way AVOVA, and Tukey HSD Post-hoc Test (*p* < 0.05) to find the variation between various groups. The type of material and the medium of storage (Dry, DW, Saliva, Ethanol) were used as independent factors. The results are presented as mean values (SD).

## 3. Results

**Figure 2:**
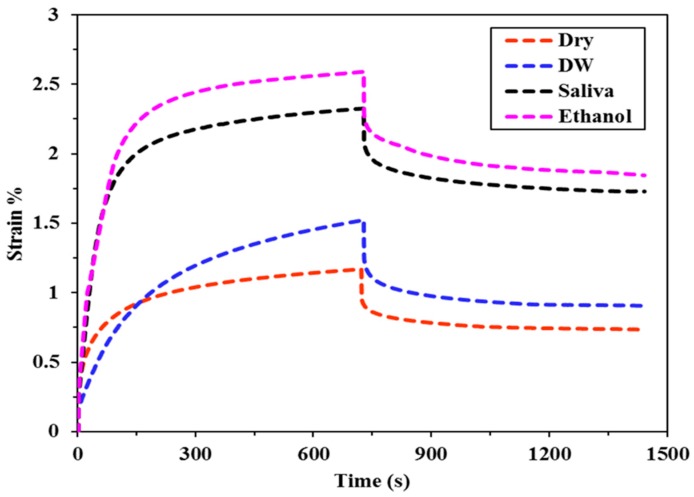
Creep deformation and recovery of FBF in various food simulating organic solvents (FSOS).

**Figure 3:**
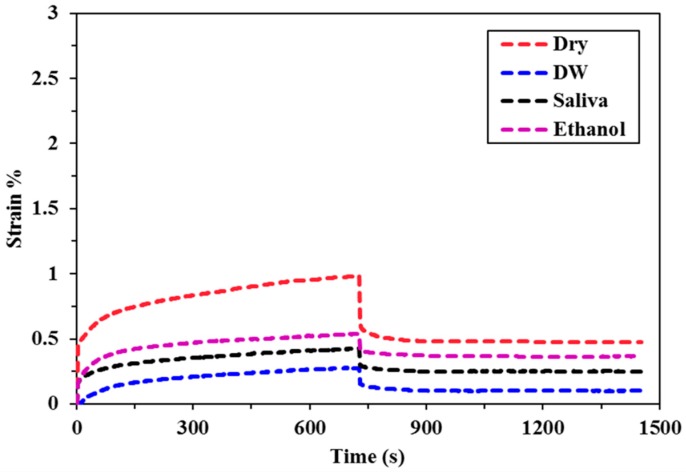
Creep deformation and recovery of the conventional material (GR) in various FSOS.

**Figure 4:**
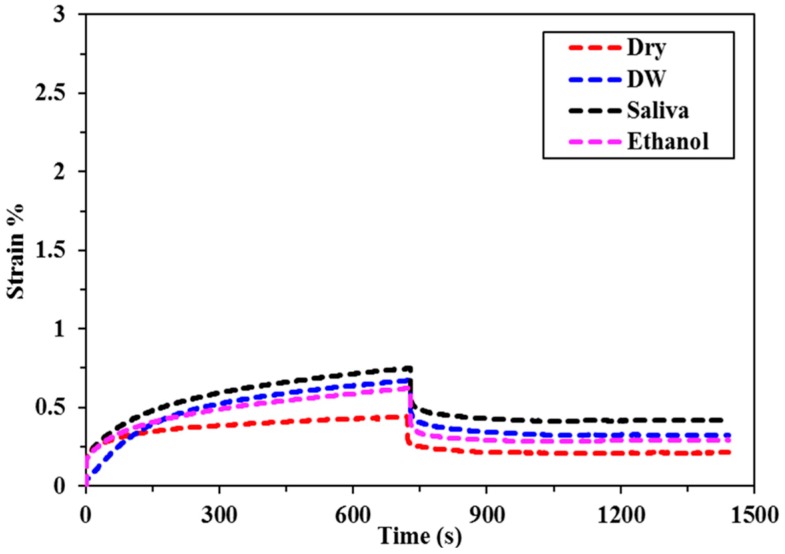
Creep deformation and recovery of SF2 in various FSOS.

**Figure 5:**
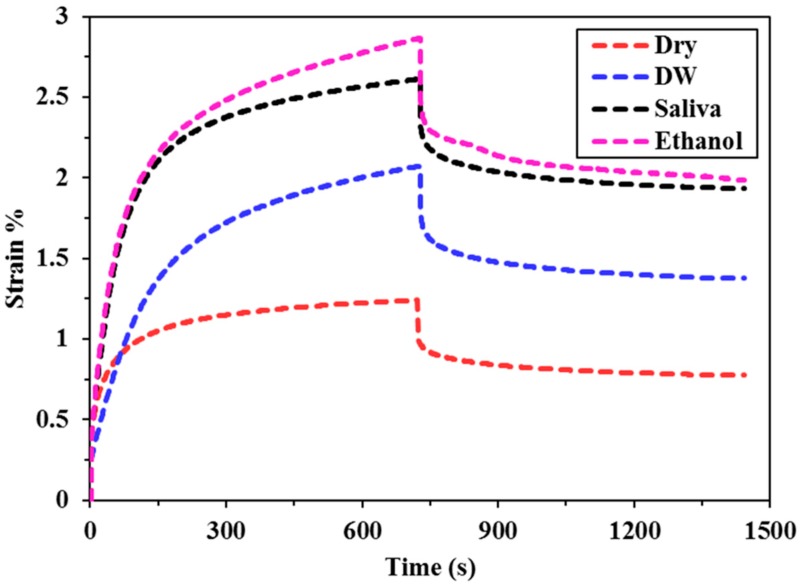
Creep deformation and recovery of TNCBF in various FSOS.

**Figure 6:**
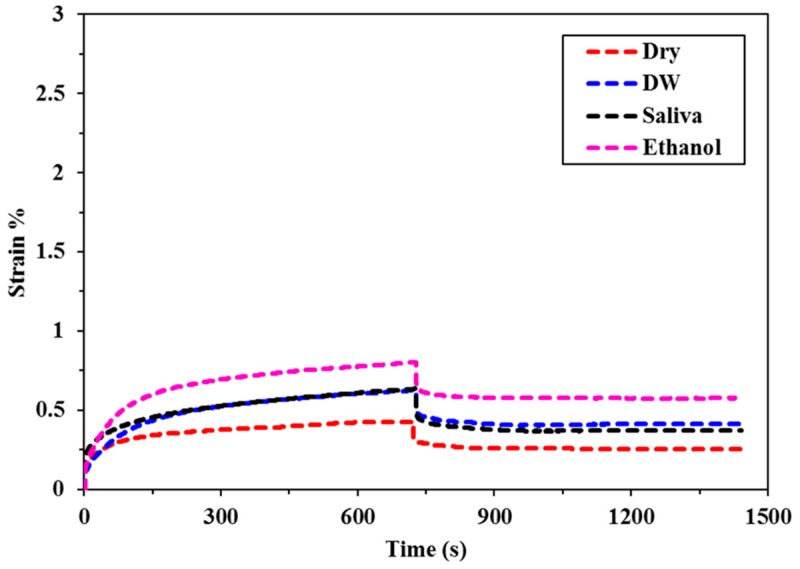
Creep deformation and recovery of XF in various FSOS.

**Figure 7:**
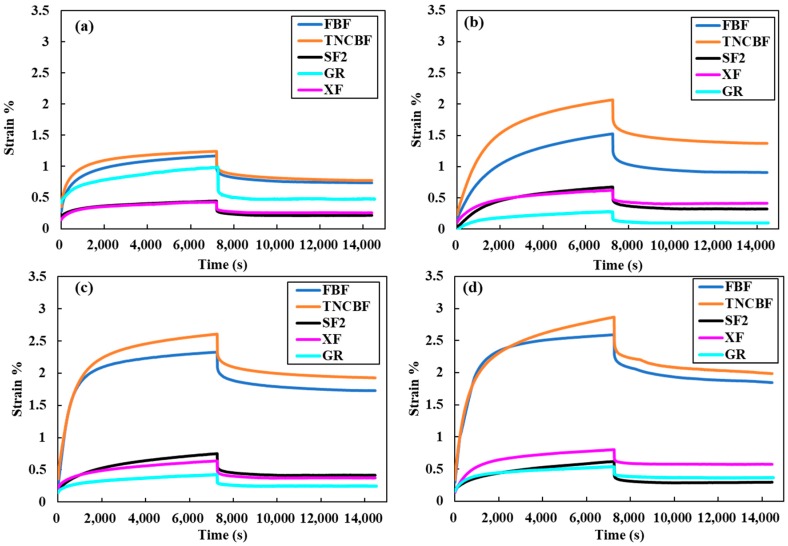
Creep deformation and recovery behavior of different Bulk fill composites in same solvent: (**a**) dry: control group; (**b**) distilled water; (**c**) artificial saliva; and, (**d**) absolute ethanol.

The creep deformation and recovery profiles for all of the bulk fill composites specimens immersed in different food simulating organic solvents (FSOS) and dry form are depicted in Figure 2, Figure 3, Figure 4, Figure 5 and Figure 6. Figure 7a–d shows the creep and recovery behavior of the various bulk fill composites in the same FSOS.

The mean values and standard deviation of maximum creep strain for all the groups in different food simulating solvents are given in Table 3. A maximum creep value (2.87) is obtained for TNCBF in ethanol while the conventional material (GR) demonstrated a minimum creep (0.28) value. All of the materials exhibited low % creep in the dry form than in the wet form except GR which showed the highest creep value in the dry form. For the maximum creep strain in all the mediums, TNCBF demonstrated significantly high creep strain (1.24 in Dry form to 2.87 in Ethanol) as compared to all of the other composite materials, except FBF, which showed no significant difference. SF2 and XF followed by GR had their least values of maximum creep in DW and Saliva, but in ethanol, XF displayed higher creep value (0.80) than the other two groups. The highest creep value was obtained for TNCBF in ethanol followed by FBF. All the composite materials resulted in an increase in the maximum creep value after immersion in FSOS. On the other hand, it was interesting to find that GR exhibited the highest maximum creep value (0.98) in the dry form, which decreased after the immersion in the FSOS. However, in terms of a permanent set, GR revealed higher values after immersion in the FSOS and a lower value in the dry form (Table 4). Similar trends were observed for permanent set % (Table 4) in the case of TNC BF and FBF, showing their highest permanent set values, while SF2 and XF showed their lowest permanent set values. With regard to maximum recovery (%), SF2 resulted in highest creep recovery, while TNCBF demonstrated the least creep recovery, as shown in Table 4 and Table 5.

### 3.1. Creep Strain

The statistical analyses of the results (Table 3) revealed that the creep strain values for SF2 and XF (SF2: 0.44 to 0.75, XF: 0.43 to 0.80) are significantly lower than TNCBF and FBF (TNC BF: 1.24 to 2.87, FBF: 1.17 to 2.59). In the dry form, SF2 and XF showed lower creep strain in comparison with the conventional material. However, after immersion in storage medium, the creep strain that was recorded for conventional material was lower than SF2 and XF. TNCBF showed the highest creep values in dry form, as well as in all of the storage mediums (1.24 in dry from; 2.07 in DW; 2.61 in saliva; and, 2.87 in ethanol), followed by FBF, which had the highest creep (2.59) after immersion in ethanol. TNCBF showed the highest creep value in absolute ethanol (2.87%), while the lowest creep value occurred with GR in distilled water (0.28%). Regarding SF2 and XF, both showed comparable values. The lowest creep value of XF was in the dry medium (0.43%), while the lowest values in DW, saliva, and ethanol were found in GR (0.28%, 0.43%, and 0.54%, respectively).

### 3.2. Recovery Strain

In the dry medium, the highest recovery strain value was observed in GR (0.93%), while the lowest value was recorded in XF (0.32%). In DW, the highest recovery strain was found in TNCBF (0.93%), whereas, the lowest value was recorded in GR (0.18%). In saliva medium, the highest recovery strain was seen in TNCBF (1.04%), while the lowest value was recorded in GR (0.33%). In ethanol medium, the highest recovery strain was found in TNCBF (1.22%), whereas, the lowest value was noted in GR (0.32%) (Table 6).

The maximum percentage of creep recovery was found in SF2 and GR in dry groups (94.59% and 94.14%, respectively). While the lowest percentage of creep recovery was found in FBF in saliva groups (35.88%). In dry medium, the lowest percentage of creep recovery value was observed in FBF (61.14%). In DW, the highest percentage of creep recovery value was noticed in GR (64.19%), while the lowest was recorded in TNCBF (45.10%). The highest percentage of creep recovery value for GR in saliva was 76.67%. In ethanol medium, the highest percentage of creep recovery value was found in SF2 (79.35%), whereas the lowest value that was achieved in FBF (40.10%) (Table 5).

### 3.3. Permanent Set

In the dry medium, the lowest permanent set was found in SF2 (0.02%), while the highest was recorded in FBF (0.45%). In DW and saliva mediums, TNCBF showed the highest permanent set values (1.14% and 1.58%, respectively), whereas GR recorded the lowest permanent set values (0.09% and 0.10%, respectively). In ethanol medium, the highest permanent set value was found in TNCBF (1.65%), while the lowest permanent set value was in SF2 (0.13%) (Table 4).

## 4. Discussion

In this study, five commercial resin based composites were immersed in food-simulating solvents of increasing solvent power to evaluate their viscoelastic stability. It is believed that the surface microhardness of dental composites may be remarkably influenced by both water absorption and the contact time with the aqueous media [23]. Thus, water has a vital role in the chemical degradation of resin composites and it affects the composite in many ways [24]. For instance, water may behave in a similar way like a weak acid that can lead to erosion (pathological loss of dental hard tissues) of filler particles and dissolution or elution of monomer [25]. The aggregation of water at the filler matrix interface either accelerate the fragmentation of inorganic particles or cause the slow promotion of the preexisted superficial flaws. The dissolution or elution of leachable components of composite resins, mainly inorganic ions or filler particles, may present, at short or long period, a deleterious effect in the polymeric network of the material, thus modifying its structure both physically and chemically [23]. The rationale for the application of saliva as a storage medium was to replicate the oral environment, which is usually weakly basic in nature. On the other hand, ethanol is a food-simulating solvent, which is employed to expose the composite to the extreme dietary conditions. In addition, they demonstrate increasing powers of plasticization and solubilization, reacting on the resin phase of composites that represent another challenge to the viscoelastic stability for that particular phase [10]. Overall, the bulk-fill composites that were immersed in different food-simulating solvents showed a statistical difference in creep strain and recovery values. Based on the results obtained, the null hypothesis was rejected.

It is apparent from the figures (Figure 2, Figure 3, Figure 4, Figure 5 and Figure 6) that specimens in different media exhibited almost similar trends with two phases in each creep strain and recovery. Upon loading, a quick elastic deformation occurred, followed by a time-dependent, slower viscoelastic deformation, which is recognized as the creep. After the removal of the load, a quick recovery took place, which followed a time-dependent, viscoelastic recovery. In the case of inadequate recovery, the specimens experienced a permanent set.

Among all of the examined materials, SF2 and XF exhibited the most favorable outcomes in the presence of all the storing media (Figure 4 and Figure 6, respectively). Furthermore, they were found to be close to the conventional resin-composite in terms of strain and recovery percentages. As per the results of the statistical analysis, both SF2 and XF exhibited lower creep deformation and higher creep recovery. However, no significant difference has been found in the maximum creep strain of SF2 and XF. This may be attributed to the high filler content of SF2 and XF (83.5%, 86%, respectively) as compared to TNCBF and FBF (78% and 76.5%, respectively). Increase in fillers loading may restrict molecular mobility and their interaction with the resin, which may result in greater creep resistance [26]. The decrease in the creep strain may be ascribed to the rise in the resin stiffness due to the reduction of free volume and the chains mobility restriction [27,28]. In addition to the impact of particulate filler, another prominent feature is the difference in the resin composition of these composites, which may lead to the significant difference in their mechanical properties. Together with the influence of higher filler content, the presence of base monomers that are structurally rigid, like bisphenol glycidyl dimethacrylates (Bis-GMA) or urethane dimethacrylates (UDMA), also contribute to improve the resistance against plasticizing effect when SF2 and XF undergo high stresses [29,30]. Thus, the lower values of creep strain, as well as permanent set and higher values of percentage creep recovery of SF2 and XF in comparison with FBF and TNCBF may be attributed to the combined effect of filler content and the presence of a structurally rigid base monomer. A study that was conducted by Papadogiannis et al. showed that Sonic Fill-1 and XF exhibited 100% recovery after 50 h of unloading [4]. However, in the current study, the creep recovery percentage after 2 h for SF-2 ranged from 59.03 to 94.59 and for XF from 45.31 to 73.99. The highest creep strain and permanent set was observed in case of TNCBF and FBF due to the low filler content (78% and 76.5%, respectively) and smaller filler’s size. On the other hand, SF2, XF and GR showed better creep resistance and percentage of creep recovery mainly because of the higher filler content (83.5%, 86% and 87%, respectively). According to the literature, XF and SF frequently showed higher mechanical strength characteristics, while FBF often showed poor mechanical strength [31]. The results that were obtained from the current study support the argument from the literature and are in line with the previous studies. Thus, it can be predicted that SF2 and XF will render promising resistance to mechanical stresses. Thereupon, the susceptibility to fracture will be reduced for these materials and they will have a restoration with a long-term durability [4,11]. On the other hand, FBF and TNCBF exhibited the highest creep deformation and permanent set upon storing in the food-simulating solvents (Figure 2 and Figure 5, respectively). The improved viscoelastic properties of bulk-fill resin-composites would encourage the dentists to employ the bulk-filling method. Precisely speaking, ethanol and saliva had significant adverse effects in terms of creep strain, strain recovery, and permanent set. The susceptibility of composite resin to deformation by the solvent can be explained on the basis of diffusion capability of solvent and the formation of a bond with polymer chains by interchanging the inter chain secondary bonds. Thereupon, food-simulating solvents make polymer chains entanglement vulnerable and enhance the dissolution of residual monomers entities. However, these solvents are not capable of harming primary covalent crosslinking; therefore, polymer molecules are not carried away by the solvents [32]. In fact, the dissolution of a material in a particular solvent is governed by their relative polarities: polar substances are likely soluble in polar solvents, while the nonpolar substances are likely to be soluble in nonpolar solvents [33]. Therefore, the variation of creep behavior of different materials in ethanol and water can be associated with the difference in their relative polarities.

It is noteworthy that the creep deformation of all the examined resin-composites increased with food-simulating solvent storage. Previous studies have shown that the viscoelastic stability, expressed by creep parameters, is mostly dependent on composition and storage-solvents [10]. All of the studied “bulk-fill” composites stored in food-simulating liquids had both higher creep strain and permanent set than the dried one. Furthermore, these materials also had lower percent creep recovery as compared to dry materials. The effect of food-simulating solvents on the SF2 and XF was almost comparable. However, they affect adversely in case of TNCBF and FBF, in particular when they were stored in ethanol and saliva. This may be due to the fact that fluid absorption by resin-composites causes the deterioration of strength, as well as composite stiffness [34]. Water is known for the chemical degradation of composite, leading to the hydrolysis reaction and swelling of material provided that the filler particles are unsilanated [24,35]. The absorbed water and moisture from saliva induce peeling stress, plasticizing effect in the structure, debonding of filler from the matrix, and altogether eventually leading to enhanced creep formation [17,36]. Furthermore, the rise in creep deformation of composites in ethanol may be attributed to the presence of some hydrophilic monomers [37,38]. Interestingly, the bulk-fill composites exhibited an acceptable creep deformation, within the range that is shown by conventional resin-composites. Since all of the specimens that are immersed in the food-simulating liquids exhibited a higher creep strain and permanent set, and the lowest percent of creep recovery, in comparison with the dry samples, it can be concluded that samples after storing into food-simulating solvents are more prone to the deteriorating effect. Based on the results and discussion, the present study can be extended for prolonged storage time to evaluate the creep deformation and recovery of several types of composites. It is likely that the longer storage time in food-simulating liquids would cause more matrix dissolution, thus decreasing the mechanical properties of the composites.

## 5. Conclusions

Food simulating liquids had a considerable influence on bulk-fill dental composites in terms of creep strain and recovery. However, bulk-fill resin-composites exhibited an acceptable creep strain and recovery, and being within the range of other conventional resin-composites. The immersion of bulk-fill composites in the food-simulating had exacerbated the creep behavior of the materials and they had higher creep deformation in comparison with the dry material. The rise in creep deformation may be associated with the swelling, peeling stress, and plasticizing effect in the structure. SF2 and XF exhibited higher creep resistance as a result of the presence of higher filler content (83.5% and 86%, respectively) and had a comparable percentage of creep recovery to the conventional materials. In addition, these materials also presented lower values of the permanent set. On the other hand, FBF and TNCBF demonstrated the highest creep deformation and permanent set upon immersion in the food-simulating solvents, particularly when they were immersed in ethanol and saliva. Thus, it can be concluded that samples after immersing in food-simulating solvents are more prone to deteriorating effects.

## Figures and Tables

**Table 1:** Specifications of the studied materials.

Material	Code	Type	Manufacturer Increment Thickness (mm)	Matrix	Filler (wt %)	Manufacturer
Tetric N-ceram Bulk Fill	TNCBF	Bulk fill	4	Bis-GMA, Bis-EMA, UDMA	78	Ivoclar Vivadent AG, Schaan, Liechtenstein
FiltekTM Bulk Fill	FBF	Bulk fill	4	AUDMA, AFM, DDDMA, UDMA	76.5	3 M ESPE GmbH, Seefeld, Germany
SonicFillTM2	SF2	Sonic-activated, bulk fill	5	Bis-GMA, TEGDMA, Bis-EMA, SIMA	83.5	Kerr Corp, Orange, USA
X-tra fil	XF	Bulk fill	4	Bis-GMA, UDMA, TEGDMA	86	Voco GmbH Cuxhaven, Germany
Grandio	GR	Nano-Hybrid	2	Bis-GMA, TEDMA, UDMA	87	Voco GmbH Cuxhaven, Germany

**Table 2:** Ingredients of artificial saliva.

Ingredients	Concentration (G/100 ML)
KH_2_PO_4_	0.3402
Na_2_HPO_4_	0.4450
HKCO_3_	1.5017
NaCl	0.5844
MgCl_2_ + 6H_2_O	0.0305
Citric Acid	0.5224
CaCl_2_	0.2205

**Table 3:** Max creep strain %.

Materials Code	Dry	Dw	Saliva	Ethanol
FBF	1.17 (0.10) a, c, A	1.52 (0.11) a, A	2.33 (0.15) a, B	2.59 (0.08) a, B
SF2	0.44 (0.07) b, A	0.67 (0.08) b, A	0.75 (0.13) b, A	0.62 (0.11) b, c, A
XF	0.43 (0.14) b, A	0.62 (0.16) b, A	0.64 (0.10) b, A	0.80 (0.14) b, A
TNC BF	1.24 (0.05) a, A	2.07 (0.19) c, B	2.61 (0.09) a, C	2.87 (0.18) a, C
GR	0.98 (0.03) c, A	0.28 (0.07) d, B	0.43 (0.05) c, B, C	0.54 (0.06) c, C

The same superscript small letters indicate no significant differences (columns) (*p* < 0.05). The same superscript capital letters indicate no significant differences (rows) (*p* < 0.05).

**Table 4:** Permanent set %.

Materials Code	Dry	Dw	Saliva	Ethanol
FBF	0.45	0.73	1.49	1.55
SF2	0.02	0.28	0.24	0.13
XF	0.11	0.31	0.17	0.44
TNC BF	0.42	1.14	1.58	1.65
GR	0.06	0.09	0.10	0.22

**Table 5:** Percentage of creep recovery %.

Materials Code	Dry	Dw	Saliva	Ethanol
FBF	61.14	51.91	35.88	40.10
SF2	94.59	59.03	67.91	79.35
XF	73.99	50.20	72.59	45.31
TNC BF	66.24	45.10	39.74	42.52
GR	94.14	64.19	76.67	60.06

**Table 6:** Max recovery strain %.

Materials Code	Dry	Dw	Saliva	Ethanol
FBF	0.72 (0.12) a, A	0.79 (0.01) a, A	0.83 (0.10) a, A, B	1.04 (0.07) a, B
SF2	0.42 (0.05) b, A	0.39 (0.04) b, A	0.51 (0.09) b, A	0.49 (0.18) b, A
XF	0.32 (0.10) b, A	0.31 (0.11) b, c, A	0.46 (0.13) b, A	0.36 (0.08) b, A
TNC BF	0.82 (0.09) c, a, A	0.93 (0.14) a, A	1.04 (0.06) c, A, B	1.22 (0.11) a, B
GR	0.93 (0.07) c, A	0.18 (0.03) c, B	0.33 (0.09) b, B	0.32 (0.03) b, B

The same superscript small letters indicate no significant differences (columns) (*p* < 0.05). The same superscript capital letters indicate no significant differences (rows) (*p* < 0.05).
